# Analysis of variola virus molecular evolution suggests an old origin of the virus consistent with historical records

**DOI:** 10.1099/mgen.0.000932

**Published:** 2023-01-09

**Authors:** Diego Forni, Cristian Molteni, Rachele Cagliani, Mario Clerici, Manuela Sironi

**Affiliations:** ^1^​ IRCCS E. MEDEA, Bioinformatics, Bosisio Parini, Italy; ^2^​ University of Milan, Milan, Italy; ^3^​ Don C. Gnocchi Foundation ONLUS, IRCCS, Milan, Italy

**Keywords:** molecular dating, population structure, smallpox, Variola virus

## Abstract

Archaeovirology efforts provided a rich portrait of the evolutionary history of variola virus (VARV, the cause of smallpox), which was characterized by lineage extinctions and a relatively recent origin of the virus as a human pathogen (~1700 years ago, ya). This contrasts with historical records suggesting the presence of smallpox as early as 3500 ya. By performing an analysis of ancestry components in modern, historic, and ancient genomes, we unveil the progressive drifting of VARV lineages from a common ancestral population and we show that a small proportion of Viking Age ancestry persisted until the 18th century. After the split of the P-I and P-II lineages, the former experienced a severe bottleneck. With respect to the emergence of VARV as a human pathogen, we revise time estimates by accounting for the time-dependent rate phenomenon. We thus estimate that VARV emerged earlier than 3800 ya, supporting its presence in ancient societies, as pockmarked Egyptian mummies suggest.

## Data Summary

All sequence data for strains used in this study were retrieved from NCBI. The accession IDs are provided in Table S1.

**Impact Statement**

Now eradicated, smallpox was one of the most devastating human diseases, causing the death of at least 300 million people in the twentieth century. Humans were the only known host of variola virus, but the time-frame of its emergence in our species has been a matter of debate. Specifically, molecular dating suggested a relatively recent origin, whereas historical sources indicated the presence of VARV in ancient societies. By applying population genetic methods, we analysed the ancestry components in modern and historic VARV genomes, and we found a progressive drifting of VARV lineages from a common ancestral population. By accounting for the common observation that rates of viral evolution scale negatively with the time-frame of measurement, we obtained estimates of VARV emergence that are over 2000 years older than previous ones. Thus, out data settle a controversy and provide novel insight into the origin and evolution of one of the most historically relevant human pathogens.

## Introduction

Variola virus (VARV), the causative agent of smallpox, represented a major cause of death during human history [1]. Vaccination against smallpox was pioneered by Edward Jenner in 1796. Although the practice spread rapidly, at the end of the nineteenth century the disease was still endemic in Europe and in many other areas [1]. Intensification of the vaccination campaign during the twentieth century led to smallpox eradication in 1980 [1]. At that time, two modern VARV (mVARV) lineages were circulating, P-I and P-II, the latter including alastrim (or variola minor) strains, characterized by much lower severity [2].

VARV is a member of the *Orthopoxvirus* genus (family *Poxviridae*), which includes several extant viruses that infect humans and other mammals. One of these viruses, monkeypox virus (MPXV) causes a disease similar to, but less severe than smallpox [3]. Once considered a rare zoonotic disease, monkeypox frequency has been increasing in the last few years, culminating in an ongoing worldwide outbreak (https://www.who.int/emergencies/situations/monkeypox-oubreak-2022). The *Orthopoxvirus* genus also includes vaccinia virus (VACV), which was used as a vaccine during the smallpox eradication campaign [1]. However, the two orthopoxviruses most closely related to VARV are camelpox virus (CMLV) and taterapox virus (TATV, which infects gerbils).

The time-frame of VARV emergence as a human pathogen has been a matter of debate. Whereas some historical sources suggest that the disease was present in Egypt and Asia as early as 3 500 years ago [4], molecular dating analyses indicated a more recent origin for the virus. Specifically, the sequencing of ancient VARV genomes (aVARV) revealed that viral strains that were transmitted during the Viking Age (600 to 1050 CE) went extinct [5]. This was also the fate of viral lineages that were sequenced from historical remains of the seventeenth and eighteenth centuries (historic VARV, hVARV) [6, 7]. Molecular dating indicated that VARV lineage extinction occurred before the initiation of vaccination campaigns [5–7].

It is increasingly recognized that molecular dating can be affected by the time-dependent rate phenomenon (TDRP) - i.e., the negatively scaling of estimated rates of viral evolution with the time-frame of measurement [8, 9]. The TDRP has little consequences for time estimates of recent events, but can profoundly affect estimates of those that occurred in a distant past [8]. Here, we analysed modern, historic, and ancient VARV genomes to infer ancestry components and to re-assess the time-frame of VARV emergence with a TDRP-aware method.

## Methods

### Alignment, nucleotide diversity, and recombination

Complete or almost complete sequences of modern VARV genomes were downloaded from the NCBI database. Samples of historical/ancient remains were retrieved from previously published works [5–7, 10]. This generated a dataset of 56 viral genomes, which were obtained with different methods and coverage (Table S1). Whole genomes (without repeat trimming) were aligned using MAFFT (v.7.475) with default parameters [11].

Nucleotide diversity was analysed by calculating θ_W_ (the scaled number of segregating sites [12]) and π (the average number of pairwise differences [13]). The site frequency spectrum statistic Tajima’s D [14] was also calculated. All analyses were performed in the R environment using the POP-GENOME package [15].

Recombination analysis was performed using the 3SEQ Recombination Detection Algorithm [16, 17] with default parameters. We accepted events with a corrected *P*-value <0.01. Breakpoints were mapped based on the 3SEQ output using the extremes of the inferred ranges.

### Linkage disequilibrium and population structure

Population structure analysis was performed by considering only biallelic parsimony-informative (PI) sites. Biallelic sites, each with a minimum frequency of two, in those genomic positions where at least 50 % of sequences had non-missing information were selected, generating a list of 4013 variants.

The LIAN software (v.3.7) [18] was applied to evaluate the level of linkage disequilibrium (LD) in the dataset. This tool tests for independent assortment by computing the number of loci at which each pair of haplotypes differs. The interpretation of LD was associated to the standardized index of association (I_A_^S^). LIAN estimated a I_A_^S^=0.294 (*P*-value<0.001, significance assessed by 1000 Monte Carlo simulations), a value indicating moderate LD (zero means linkage equilibrium) and therefore allowing the application of population structure analyses as implemented in the STRUCTURE (v.2.3.4) suite [19]. This tool divides the whole population into K subpopulations characterized by a set of allele frequencies at each locus [19]. We first estimated the allele frequency spectrum parameter by running STRUCTURE with K=1, as suggested [20]. This λ parameter was estimated to be equal to 0.487 and then the linkage model with correlated allele frequencies was run [20] for K from 1 to 12. In particular, for each K, ten runs were performed with a MCMC chain length of 500 000 iterations and 50 000 burn-in and map distances were set equal to PI site physical distances. To obtain more accurate inferences in spite of the different representation of ancient and modern genomes, as well as of P-I and P-II sequences, we used an ancestry prior that allows source populations to contribute differentially to the pooled sample of individuals [21].

To choose the optimal K, the Evanno’s method [22] in the HARVESTER tool [23] were applied. Finally, the CLUMPAK [24] software was used to combine replicate runs for the same K.

The linkage model allows us to estimate the amount of drift that each K subpopulation experienced from a common ancestral population, which is characterized by a set of estimated allele frequencies.

This drift is quantified by the F parameter [20], calculated for the optimal K.

### Molecular dating

By taking advantage of a heterochronous dataset also composed by ancient data and by the presence of a temporal signal already demonstrated in previously published works [5, 7], we performed a time estimate phylogenetic reconstruction. In particular, we wished to correct the already proposed molecular dating of the VARV phylogeny [5, 7] by accounting for the time dependent rate phenomenon (TDRP) [25].

Thus, we applied a recently developed method (PoW, prisoner of war, model) [8] on our VARV dataset plus the complete genomes of related viruses taterapox (TATV, NC_008291) and camelpox (CMLV, NC_003391). Two Czech museum specimens were excluded from the analysis due to their controversial date estimates [7, 10, 26].

Using two large non-recombining regions (Fig. S1) for the VARV/TATV/CMLV and VARV only datasets, we constructed the distribution of ultrametric distance trees with the BEAST (v.1.10.4) software [27] by running a hundred million iterations MCMC chain, sampled every 10 000 steps, using a strict clock model, a constant size population tree prior, and the HKY85 substitution model, as suggested [8].

After discarding the 10 % burn-in, the run was checked with Tracer (v.1.7.1) [28] for having ESS (effective sampling size) values>200, thus generating a set of 9000 distance trees. We then converted these distance trees to time trees using the median substitution rate estimated by Mühlemann and co-workers [5] (i.e. 5.16×10^−6^ s/s/y) and by applying the PoW model [8]. Finally, using TreeAnnotator [27]*,* we generated a TDRP-correct consensus tree, which was visualized with FigTree (http://tree.bio.ed.ac.uk/).

## Results and discussion

**Fig. 1.**
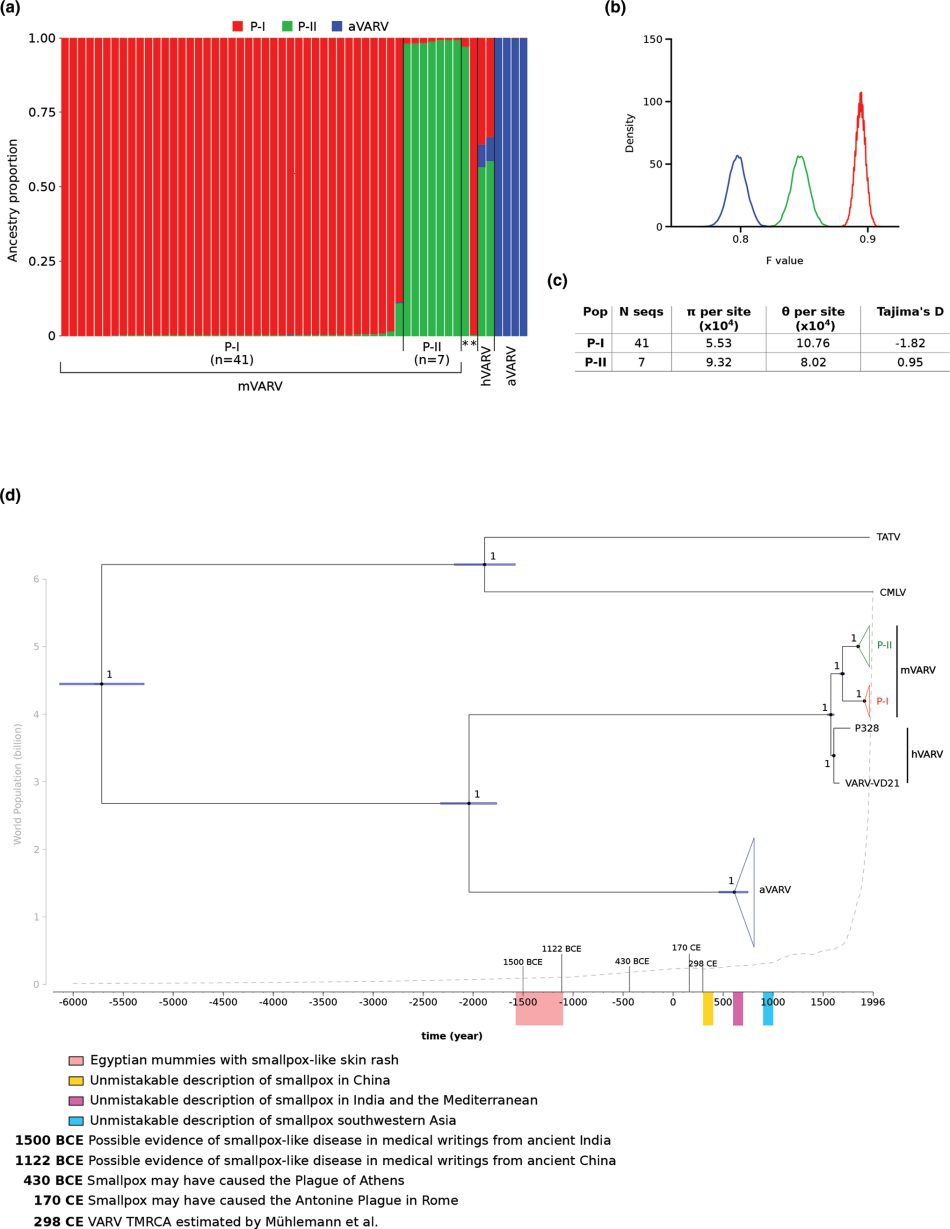
Population structure and molecular dating of VARV genomes. (**a**) Bar plot representing the proportion of ancestral population components. Each vertical line represents a VARV genome and it is coloured by the proportion of sites that have been assigned to each population by STRUCTURE. Asterisks denote two samples (LT706529 and LT706528) with controversial dates [7, 10] (**b**) Distribution of F values for the three populations [colours as in (**a**)]. (**c**) Nucleotide diversity estimators and Tajima’s D [14] for P-I and P-II clades. (**d**) Dated maximum credibility tree re-scaled after the TDRP correction. Branch lengths represent evolutionary time in years and a timescale grid is shown at the tree base, where some relevant historical events are highlighted [4]. For each node, bars indicates 95 % HPD intervals of node ages and the number indicates bootstrap support. World population size at different time points is reported as a grey dashed line (https://ourworldindata.org/).

Like all orthopoxviruses, VARV has a long (~185 Kbp) double-stranded DNA genome. This allows the application of approaches that use multilocus genotype data to analyse ancestry and admixture patterns. We thus generated an alignment of 56 VARV genomes (four aVARV, two hVARV, 48 mVARV, and two samples with controversial dating, Table S1) and we analysed 4013 parsimony-informative markers. These were used to evaluate the strength of linkage disequilibrium, which resulted moderate. This is unlikely to be due to recombination, which, in agreement with previous reports, we found to be relatively uncommon in the VARV dataset (Fig. S1, available in the online version of this article) [2, 5]. Rather, the moderate association among sites is most likely due to other factors such as mutation and unequal selective pressure along the genome. In any case, the moderate LD we detected warrants the application of models implemented in the STRUCTURE programme [19]. STRUCTURE relies on a Bayesian statistical model for clustering genotypes into populations without prior information on their genetic relatedness or sampling date [19]. The programme can identify distinct subpopulations (or clusters, K) that compose the overall population and assigns each genome to one or more of those clusters. We applied the linkage model with correlated allele frequencies [19, 20] and we used an ancestry prior that allows source populations to contribute differentially to the pooled sample of individuals [21]. The ΔK method [22] estimated the best number of subpopulations to be equal to three (Fig. S2). Analysis of ancestry components clearly separated aVARV, P-I, and P-II genomes, with only evidence of minor subpopulation sharing in one P-I sequence (strain India-1967). Conversely, hVARV genomes showed the contribution of all ancestral populations, although the aVARV component was the least abundant (Fig. 1a). Two samples with controversial sampling dates [10] clustered with P-I and P-II lineages, in accordance with phylogenetic analyses [7]. By estimating the F parameter, which represents a measure of genetic differentiation between populations, the linkage model also provides information on the level of drift of each subpopulation from a hypothetical common ancestral population. In line with sampling dates, the lowest drift was observed for the aVARV component. However, P-II had intermediate drift between aVARV and P-I components (Fig. 1b).

Overall, these results portray a richer picture of VARV evolution, beyond the extinction of VARV lineages observed in phylogenetic analyses. Identification of ancestry proportions unveils the progressive drifting of VARV lineages from a common ancestral population and indicates that a small fraction of Viking Age ancestry persisted until the eighteenth century. Results herein also show that the P-I lineage (variola major) experienced a bottleneck that was more extreme than that of P-II (variola minor) (Fig. 1b). This conclusion was also confirmed by the calculation of nucleotide diversity [12, 13] and Tajima’s D [14] (Fig. 1c). However the small sample size for the P-II lineage suggests caution in the interpretation of these estimators. It is also worth noting that STRUCTURE estimates how much each subpopulation drifted from an ancestral population, but it does not provide direct information about the level of similarity of two or more subpopulations. In this respect, it largely differs from classic phylogenetic reconstruction and provides complementary information.

In order to analyse which aVARV variants persisted in hVARV sequences, we exploited the site-by-site inference in STRUCTURE, which allows population-of-origin assignment for individual variants. The analysis identified 47 variants with very high probability (>0.90) of an aVARV ancestry and present in hVARV genomes. Nine of these are non-synonymous changes in five genes (Table 1, Fig. S3), some of which may encode proteins (Q1L, ankyrin repeat- and kelch-like containing proteins) involved in immunomodulation and virulence [29–31]. However, most of these proteins are poorly characterized.

**Table 1.** Nonsynonymous changes in aVARV samples that persisted into the hVARV genomes

Gene^∗^		Descriptions	Position^∗^	Amino acid changes
Q1L	VARVgp053	Homolog of vaccinia O1L; may activate the ERK1/2 signalling pathway and increase virulence [31]	329	R (aVARV and hVARV), K (mVARV)
A28L	VARVgp132	Structural protein of the mature virion (viral A-type inclusion protein)	90	R (aVARV and hVARV), G (mVARV)
			609	V (aVARV and hVARV), A/T (mVARV)
			612	V (aVARV and hVARV), A (mVARV)
J7R	VARVgp165	Kelch-like domain containing protein	17	A (aVARV and hVARV), V (mVARV)
B6R	VARVgp174	Ankyrin-repeat containing protein	494	C (aVARV and hVARV), Y (mVARV and aVARV)
B18L	VARVgp186	Protein of unknown function (DUF1231)	31	T (aVARV and hVARV), M (mVARV)
			266	A (aVARV and hVARV), T (mVARV and aVARV)
			273	T (aVARV and hVARV), A (mVARV)

*Gene nomenclature and position refer to the reference genome (NC_001611).

*aVARV*, *ancient variola virus*; *CMLV*, *camelpox virus*; *hVARV*, *historic variola virus*; *IAS*, *standardized index of association*; *LD*, *linkage disequilibrium*; *MPXV*, *monkeypox virus*; *mVARV*, *modern variola virus*; *PI*, parsimony-informative; *PoW*, *prisoner of war*; *TATV*, *taterapox virus*; *TDRP*, *time-dependent rate phenomenon*; *TMRCA*, *time to most recent common ancestor*; *VACV*, *vaccinia virus*; *VARV*, *variola virus*.

We next re-assessed the time-frame of VARV emergence by accounting for the TDRP. Specifically, we applied the recently developed PoW (prisoner of war) model [8] to convert units of branch lengths in the VARV phylogeny from substitutions/site to divergence time. Specifically, we used the median substitution rate estimated by Mühlemann and co-workers [5] (i.e. 5.16×10^−6^ s/s/y) and we applied the PoW conversion [8]. The reason why we selected this substitution rate is that Mühlemann and colleagues calculated it by using VARV sequences sampled over the same time-frame as the ones we included herein (i.e., aVARV, hVARV, and mVARV), whereas previous estimates did not include aVARV genomes (Table 2). For the dating analysis, we generated trees that also included TATV and CMLV.

**Table 2.** Comparison between node ages estimated in this work and in previous ones.

Estimate	This work (with CMLV and TATV)	This work (VARV only)	Muhlemann *et al.*	Ferrari *et al.*	Duggan *et al.*
**Mean substitution rate (s/s/y)**	5.16×10^−6^ (Mühlemann *et al.*)	5.16×10^−6^ (Mühlemann *et al.*)	5.16×10^−6^	10.67×10^−6^	8.5×10^−6^
**TMRCA**	5718 BCE	na	na	na	na
**TMRCA VARV**	2045 BCE	2767 BCE	298 CE	na	na
**TMRCA mVARV/hVARV**	1574 CE	1428 CE	1534 CE	1631/1677 CE	1616 CE
**TMRCA PI/PII**	1690 CE	1600 CE	1662 CE	1789 CE	1763 CE
**TMRCA PI**	1910 CE	1891 CE	~1900 CE	1892 CE	1909 CE
**TMRCA PII**	1847 CE	1823 CE	~1800 CE	1866 CE	1870 CE

^∗^Previous estimates are from [6, 12, 19]. na: not available.

In line with the expectation under the PoW model, the TMRCA (time to most recent common ancestor) we estimated for the mVARV and hVARV clades (448 ya, 95 % HPD: 416–482) and the mVARV TMRCA (332 ya, 95 % HPD: 310–357) were very similar to previous estimates (Fig. 1d, Table 2). However, the TMRCA of all VARV genomes was pushed back to ~4000 ya (95 % HPD: 3790–4351), and we estimated that VARV split from the TATV/CMLV lineage ~7700 ya (Fig. 1d). This latter finding does not imply that VARV entered human populations at that time, as the spill-over may have occurred at any time between the split from TATV/CMLV and the VARV TMRCA (i.e., between ~8000 and ~4000 ya). Finally, we dated the TATV/CMLV split around 3800 ya (Fig. 1d).

The substitution rate we used for the PoW model was derived by analysis of VARV sequences [5]. Because TATV and CMLV evolved in non-human hosts with distinct generation times and biological features, the rate(s) of evolution in those species or the unknown species that was the actual reservoir for VARV is unknown. We thus verified that the time estimates of the VARV phylogeny were not affected by the inclusion of the two animal viruses by repeating molecular dating for VARV sequences only. Time estimates were very similar to those obtained for the extended phylogeny and the TMRCA of all VARV amounted to ~4700 ya (95 % HPD: 4458–5093) (Table 2)*.*

The PoW model is based on the idea that different sites in a sequence evolve at different rates, depending on the level of constraint. Such constraint may be host-driven or not, and the model does not require calibration information. The PoW model was shown to accurately recapitulate the evolutionary history of mammalian foamy viruses, which co-diverged with their hosts [8, 9]*.* In the case of VARV, the time-frames we estimated are consistent with archaeological evidence of smallpox in ancient Egypt and with the presence of the disease in Asia in the second millennium BCE. A major criticism of the early existence of smallpox is the absence of any mention of it in written documents from ancient Egypt or in other early sources, such as the Old and New Testaments or the work of Hippocrates. However, the pattern of gene inactivation detected in aVARV genomes led to the suggestion [5, 32] that smallpox might have been a mild disease in the initial phases of human transmission. Thus, it might have gone unnoticed in early records. Of course, though, a number of other infectious diseases cause a rash similar to smallpox and only the sequencing of archaeological specimens will provide information on which ancient societies were affected by the disease.

## Conclusions

Overall, the time-frame of VARV and CMLV emergence, the possible presence of smallpox in ancient Egypt, and the likely circulation in gerbils of the VARV/CMLV/TATV ancestor are consistent with an origin of the human pathogen in Africa or the Middle East, as suggested [2]. The two main VARV lineages split before the onset of global smallpox vaccination, in a time that corresponds to a steep increase in human population (Fig. 1d) and to long-range human movements. It is commonly thought that, after their split, the P-I and P-II lineages underwent severe bottlenecks, as the result of increasing vaccination. Our results however show that the bottleneck was definitely more severe for P-I than for P-II, as also suggested by calculation of nucleotide diversity and Tajima’s D [14] (Fig. 1c). One possible explanation for this finding is that, because of its generally lower virulence [2], the P-II lineage maintained an endemic, low-level transmission in some geographic areas (e.g., West Africa whence it reached South America), whereas the P-I lineage represented an epidemic, highly-virulent lineage that was rapidly recognized and disposed of by control measures (e.g., isolation and quarantine) [1]. Indeed, during the twentieth century, in areas where mild and severe smallpox forms were transmitted, the former persisted much longer in the population than the latter [1]. Another, non-mutually exclusive possibility is that the two lineages were transmitted in regions where vaccination progressed with different pace and efficiency, thus resulting in different selective effects on circulating viruses. In line with this possibility, the population size of the P-I lineage was recently shown to have rapidly decreased around 1965–1970, a time when the worldwide eradication campaign intensified [33]. It should also be noticed that only seven P-II sequences were available for analysis and thus inferences about the population history for this clade should be taken with caution. Indeed, our study has limitations, which are inherent to the limited dataset of both modern and ancient VARV sequences, their biassed geographic origin, and their often complex (and unknown) passage history. Likewise, the long branches separating VARV, TATV, and CMLV underscore the extent of unsampled viral diversity for orthopoxviruses. Overall, although our results provide little insight into the ultimate origin of VARV as a human pathogen, they contribute to re-assess its temporal emergence and evolutionary trajectory.

## Supplementary Data

Supplementary material 1
