# Supplementary material for: Analysis of variola virus molecular evolution suggests an old origin of the virus consistent with historical records
Source: Microb Genom. 2023 Jan 9;9(1):mgen000932. doi: 10.1099/mgen.0.000932 (PMC9973844; doi:10.1099/mgen.0.000932)
Supplement: Supplementary material 1 [file mgen-9-932-s001.pdf]

Table S1. List of analyzed strains.

Strain name	NCBI Accession ID	Lineage	Method (coverage)
VARV_V1588*	LT706529*	-	NGS (6900x)
VARV_V563*	LT706528*	-	NGS (1700x)
VK388	LR800244	aVARV	NGS (7.7x)
VK382	LR800245	aVARV	NGS (45x)
VK281	LR800246	aVARV	NGS (5x)
VK470	LR800247	aVARV	NGS (7.5x)
seq_P328_London	P328	hVARV	NGS (14x)
VD21	KY358055	hVARV	NGS (18x)
Variola virus strain Germany 1958 Heidelberg	DQ437584	mVARV P-I	Sanger
Variola virus strain Somalia 1977	DQ437590	mVARV P-I	Sanger
India-1967	NC_001611	mVARV P-I	Subcloning and Maxam–Gilbert
Variola virus strain Korea 1947 (Lee, Masterseed)	DQ441432	mVARV P-I	Sanger
Variola virus strain China Horn 1948	DQ437582	mVARV P-I	Sanger
Variola virus strain Japan 1951 (Harper, Masterseed)	DQ441430	mVARV P-I	Sanger
Variola virus strain United Kingdom 1946 Harvey	DQ441444	mVARV P-I	Sanger
Variola virus strain Sumatra 1970 V70-228	DQ441442	mVARV P-I	Sanger
Variola virus strain Sumatra 1970 V70-222	DQ437591	mVARV P-I	Sanger
Variola virus strain India 1953 (New Delhi)	DQ441428	mVARV P-I	Sanger
Variola virus strain Japan 1946 (Yamada MS-2(A) Tokyo)	DQ441429	mVARV P-I	Sanger
Variola virus strain Japan 1951 (Stillwell, Masterseed)	DQ441431	mVARV P-I	Sanger
Variola virus strain Kuwait 1967 (K1629)	DQ441433	mVARV P-I	Sanger
Variola virus strain United Kingdom 1947 Higgins (Staffordshire)	DQ441446	mVARV P-I	Sanger
Variola virus strain United	DQ441445	mVARV P-I	Sanger

Kingdom 1946 Hinden (Middlesex)			
Variola virus strain Afghanistan 1970 Variolator 4	DQ437580	mVARV P-I	Sanger
Variola virus strain Bangladesh 1975 v75-550 Banu	DQ437581	mVARV P-I	Sanger
Variola virus strain Congo 1970	DQ437583	mVARV P-I	Sanger
Variola virus strain India 1964 7124 Vellore	DQ437585	mVARV P-I	Sanger
Variola virus strain India 1964 7125 Vellore	DQ437586	mVARV P-I	Sanger
Variola virus strain Iran 1972 2602 Tabriz	DQ437587	mVARV P-I	Sanger
Variola virus strain Nepal 1973	DQ437588	mVARV P-I	Sanger
Variola virus strain Pakistan 1969 (Rafig Lahore)	DQ437589	mVARV P-I	Sanger
Variola virus strain Syria 1972 V72-199	DQ437592	mVARV P-I	Sanger
Variola virus strain Botswana 1972 (v72-143)	DQ441417	mVARV P-I	Sanger
Variola virus strain Botswana 1973 (v73-225)	DQ441418	mVARV P-I	Sanger
Variola virus strain Bangladesh 1974 (nur islam)	DQ441420	mVARV P-I	Sanger
Variola virus strain Bangladesh 1974 (Shahzaman)	DQ441421	mVARV P-I	Sanger
Variola virus strain Bangladesh 1974 (Solaiman)	DQ441422	mVARV P-I	Sanger
Variola virus strain Congo 9 1970 (v74-227 Gispen)	DQ441423	mVARV P-I	Sanger
Variola virus strain India 1953 (Kali-Muthu-M50 Madras)	DQ441427	mVARV P-I	Sanger
Variola virus strain South Africa 1965 (102 Natal, Ingwavuma)	DQ441435	mVARV P-I	Sanger
Variola virus strain South Africa 1965 (103 T'vaal, Nelspruit)	DQ441436	mVARV P-I	Sanger
Variola virus strain Tanzania 1965 kembula	DQ441443	mVARV P-I	Sanger

Variola virus strain Yugoslavia 1972 V72-164	DQ441448	mVARV P-I	Sanger
Variola virus strain Ethiopia 1972 (Eth16 R14-1X-72 Addis)	DQ441424	mVARV P-I	Sanger
Variola virus strain Ethiopia 1972 (Eth17 R14-1X-72 Addis)	DQ441425	mVARV P-I	Sanger
Variola virus strain Somalia 1977 (V77-1252)	DQ441438	mVARV P-I	Sanger
Variola virus strain Somalia 1977 (V77-1605)	DQ441439	mVARV P-I	Sanger
Variola virus strain Sudan 1947 (Juba)	DQ441440	mVARV P-I	Sanger
Variola virus strain Sudan 1947 (Rumbec)	DQ441441	mVARV P-I	Sanger
Variola virus strain Benin, Dahomey 1968 (v68-59)	DQ441416	mVARV P-II	Sanger
Variola virus strain Guinea 1969 (005)	DQ441426	mVARV P-II	Sanger
Variola virus strain Sierra Leone 1969 (V68-258)	DQ441437	mVARV P-II	Sanger
Variola virus strain Niger 1969	DQ441434	mVARV P-II	Sanger
Variola virus strain United Kingdom 1952 Butler	DQ441447	mVARV P-II	Sanger
Variola virus strain Brazil 1966 (v66-39 Sao Paulo)	DQ441419	mVARV P-II	Sanger
Garcia-1966	Y16780	mVARV P-II	Subcloning and Sanger

* samples with controversial date estimates (see text for details). NGS: next-generation sequencing

Figure S1. (a) VARV recombination events. Unique recombination events in 54 VARV genomes (two samples with controversial dating were excluded from the analysis). Each event is shown as a line with dots representing the start and the end. The location of selected nonrecombinant region used in the analyses is shown in grey (region length: 136500 bp). Positions refer to the whole genome alignment.(b) Unique recombination events in 54 VARV genomes and the 2 outgroups strains (CMLV: NC_003391, TATV: NC_008291).The location of selected nonrecombinant region used in the analyses is shown in grey (region length: 92500 bp).

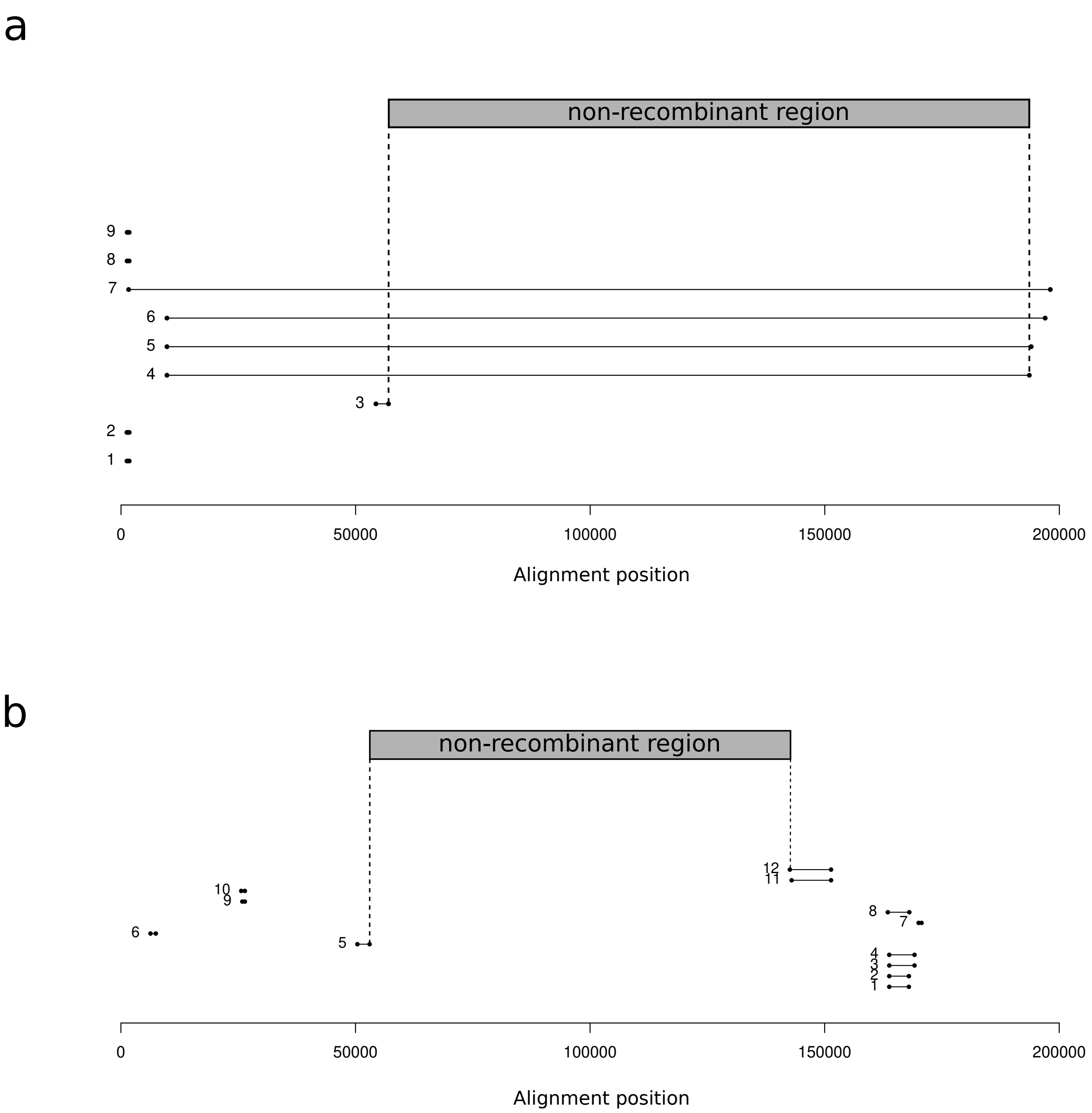

Figure S2. ΔK analysis. Identification of the optimal K for STRUCTURE analysis using the ΔK method.

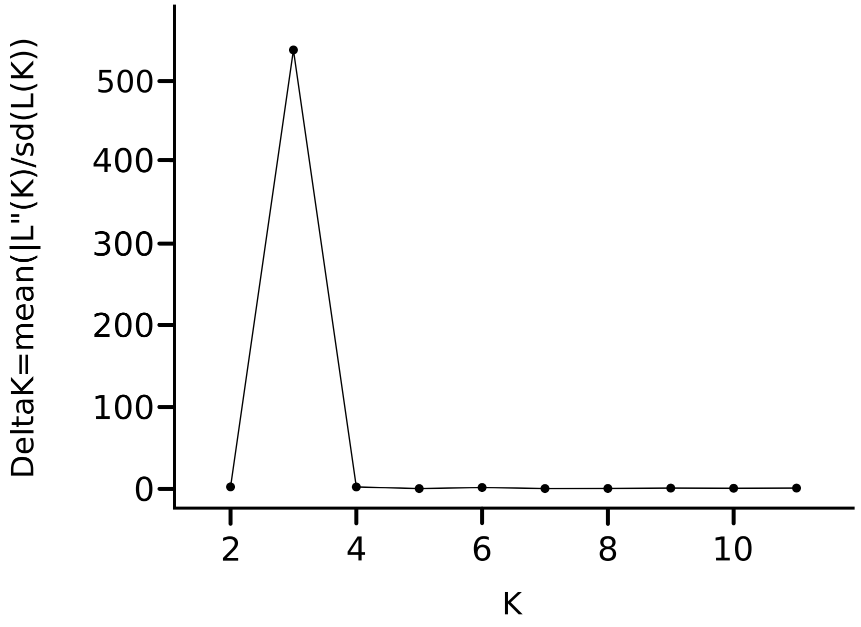

Figure S3. aVARV variants present in hVARV genomes. Amino acid alignments of the non-synonymous variants (and their surrounding regions) with very high probability (>0.90) of aVARV ancestry and present in hVARV genomes. The identified variants are highlighted and colors are as follows: red, P-I lineage; green, P-II lineage; pink, samples with controversial date estimate; purple, hVARV lineage, light blue, aVARV lineage. For P-I and P-II lineages, representative strains are shown. Positions refer to VARV reference strain (NC_001611).

VARVgp053

```
            320         329         340
DQ441448      RQITMQTGDKIRRFRRPMSLR
DQ441428      RQITMQTGDKIRRFRRPMSLR
DQ437590      RQITMQTGDKIRRFRRPMSLR
NC_001611     RQITMQTGDKIRRFRRPMSLR
X69198        RQITMQTGDKIRRFRRPMSLR
DQ441437      RQITMQTGDKIRRFRRPMSLR
DQ441447      RQITMQTGDKIRRFRRPMSLR
DQ441416      RQITMQTGDKIRRFRRPMSLR
Y16780        RQITMQTGDKIRRFRRPMSLR
DQ441426      RQITMQTGDKIRRFRRPMSLR
LT706529      RQITMQTGDKIRRFRRPMSLR
LT706528      RQITMQTGDKIRRFRRPMSLR
KY358055      RQITMQTGDRIRRFRRPMSLR
P328          RQITMQTGDRI--FRRP--LR
LR800244      RQITMQTGNRIRRFRRPMSLR
LR800247      RQITMQTGDRIRRFRRPMSLR
LR800246      RQITMQTGDRIRRFRRPMSLR
LR800245      RQITMQTGNRIRRFRRPMSLR
```

VARVgp132

```
             80         90         99      600        609 612      620
DQ441448     YKTIFNNGVDAESRINTTGK........YQQDRDTLSAVRGELEEERRR
DQ441428     YKTIFNNGVDAESRINTTGK........YQQDRDTLSAVRGELEEERRR
X69198       YKTIFNNGVDAESRINTTGK........YQQDRDTLSAVRGELEEERRR
DQ437590     YKTIFNNGVDAESRINTTGK........YQQDRDTLSAVRGELEEERRR
NC_001611    YKTIFNNGVDAESRINTTGK........YQQDRDTLSAVRGELEEERRR
DQ441437     YKTIFNNGVDAESRINTTGK........YQQDRDTLSAVRGELEDERRR
DQ441416     YKTIFNNGVDAESRINTTGK........YQQDRDTLSAVRGELEDERRR
DQ441447     YKTIFNNGVDAESRINTTGK........YQQDRDTLSAVRGELEEERRR
Y16780       YKTIFNNGVDAESRINTTGK........YQQDRDTLSAVRGELEEERRR
DQ441426     YKTIFNNGVDAESRINTTGK........YQQDRDTLSAVRGELEDERRR
LT706529     YKTIFNNGVDAESRINTTGK........YQQDRDTLSAVRGELEEERRR
LT706528     YKTIFNNGVDAESRINTTGK........YQQDRDTLSAVRGELEEERRR
P328         YKTIFNNGVDV-SRINTTGK........YQQD-DT-SVVRREL-EE-RR
KY358055     YKTIFNNGVDVESRINTTGK........YQQDRDTLSVVRRELEEERRR
LR800245     YKTIFNNGVDVESRINTTGK........YQQDRDTLSVVRRELEEERRR
LR800244     YKTIFNNGVDVESRINTTGK........YQQDRDTLSVVRRELEEERRR
LR800247     YKTIFNNGVDVESRINTTGK........YQQDRDTLSVVRRELEEERRR
LR800246     YKTI---------------K........YQQDRDTLSVVRRELEEERRR
```

VARVgp165

```
              10      17       25
DQ441448      YRSSKVIVYNTCTNSW
DQ441428      YRSSKVIVYNTCTNSW
DQ437590      YRSSKVIVYNTCTNSW
NC_001611     YRSSKVIVYNTCTNSW
X69198        YRSSKVIVYNTCTNSW
DQ441437      YRSSKVIVYNTCTNSW
DQ441447      YRSSKVIVYNTCTNSW
DQ441416      YRSSKVIVYNTCTNSW
Y16780        YRSSKVIVYNTCTNSW
DQ441426      YRSSKVIVYNTCTNSW
LT706528      YRSSKVIVYNTCTNSW
LT706529      YRSSKVIVYNTCTNSW
P328          YRSSK---YNTCTNYW
KY358055      YRSSKVIAYNTCTNSW
LR800247      YRSSKVIAYNTCTNSW
LR800244      YRSSKVIAYNTCTNSW
LR800245      YRSSKVIAYNTCTNSW
LR800246      YRSSKVIAYNTCTNSW
```

VARVgp174

```
                485         494    500
DQ441448          YGRHPSFVKYTSLDVY
DQ441428          YGRHPSFVKYTSLDVY
NC_001611         YGRHPSFVKYTSLDVY
X69198            YGRHPSFVKYTSLDVY
DQ437590          YGRHPSFVKYTSLDVY
DQ441416          YGRHPSFVKYTSLDVY
DQ441437          YGRHPSFVKYTSLDVY
DQ441447          YGRHPSFVKYTSLDVY
Y16780            YGRHPSFVKYTSLDVY
DQ441426          YGRHPSFVKYTSLDVY
LT706528          YGRHPSFVKYTSLDVY
LT706529          YGRHPSFVKYTSLDVY
P328              YGRH-SFVKCTSLDVY
KY358055          YGRHPSFVKCTSLDVY
LR800244          YGRHPSFVKCTSLDVY
LR800245          YGRHPSFVKCTSLDVY
LR800246          YGRHPSFIKYT-----
LR800247          YGRHPSFVKCTSLDVY
```

VARVgp186

```
                  25    31  35    260     266    273 277
DQ441448          YNDSFIASPEY......DLIKDVVIFKNKEMVYYL
DQ441428          YNDSFIASPEY......DLIKDVVIFKNKEMVYYL
X69198            YNDSFIASPEY......DLIKDVVIFKNKEMVYYL
DQ437590          YNDSFIASPEY......DLIKDVVIFKNKEMVYYL
NC_001611         YNDSFIASPEY......DLIKDVVIFKNKEMVYYL
DQ441416          YNDSFIASPEY......DLIKDVVIFKNKEMVYYL
DQ441437          YNDSFIASPEY......DLIKDVVIFKNKEMVYYL
DQ441447          YNDSFIASPEY......DLIKDVVIFKNKEMVYYL
Y16780            YNDSFIASPEY......DLIKDVVIFKNKEMVYYL
DQ441426          YNDSFIASPEY......DLIKDVVIFKNKEMVYYL
LT706529          YNDSFIASPEY......DLIKDVVIFKNKEMVYYL
LT706528          YNDSFIASPEY......DLIKDVVIFKNKEMVYYL
KY358055          YNDSFITSPEY......DLIKDVAIFKNKETVYYL
P328              Y-DSFI-SPEY......DLIKDVAIFKNK-TVYYL
LR800244          YNDSFITSPEY......DLIKDVAIFKNKETVYYL
LR800245          YNDSFITSPEY......DLIKDVAIFKNKETVYYL
LR800246          YNDSFITSPEY......DLIKDVTIFKNKETVYYL
LR800247          YNDSFITSPEY......DLIKDVTIFKNKETVYYL
```
